# Effect of Intraocular Lens Diameter Implanted in Enucleated Porcine Eye on Intraocular Pressure Induced by Scleral Depression

**DOI:** 10.1155/2014/586060

**Published:** 2014-03-27

**Authors:** Gaku Terauchi, Celso Soiti Matsumoto, Kei Shinoda, Harue Matsumoto, Atsushi Mizota

**Affiliations:** ^1^Department of Ophthalmology, Teikyo University School of Medicine, 2-11-1 Kaga, Itabashi-ku, Tokyo 173-8605, Japan; ^2^Matsumoto Eye Clinic, 50-2 Takagaki, Awa-cho, Awa-shi, Tokushima 771-1705, Japan

## Abstract

The effect of the diameter of an intraocular lens (IOL) implanted in enucleated porcine eyes on the intraocular pressure induced by scleral depression was investigated. Two IOLs of 6 mm and 7 mm optic diameter were implanted. The intraocular pressure (IOP) was monitored during scleral depression by a transducer placed in the midvitreous through a sclerotomy at 6 o'clock. The area under the curve (AUC) of the IOP changes from the beginning of the indentation to the point when the peripheral retinal surface was observed through the IOL optics was measured. The AUC was significantly larger in eyes with a 6 mm IOL than in eyes with a 7 mm IOL (*p* < 0.05). The IOP elevation at the endpoint was higher in eyes with the 6 mm IOL than in eyes with the 7 mm IOL. We conclude that the AUC may represent the degree of stress induced by scleral depression. The higher AUC value with the X-60 may be because of the longer distance from the peripheral retina to the edge of the IOL optics.

## 1. Introduction

Small-incision cataract surgery and microincision transconjunctival vitrectomy have allowed combining pars plana vitrectomy with phacoemulsification and intraocular lens (IOL) implantation with less time and less invasion [1, 2]. In cases where the IOL is implanted first, the fundus visibility is improved by the clear IOL. However, the edge of the optics of the IOL can interfere with the observation of the equatorial and more peripheral areas of the retina. To overcome this limited view, scleral depression is used to observe the peripheral fundus [3, 4]. Scleral depression is a useful procedure; however, considerable pressure may be required to bring the peripheral retina and vitreous base into view. Scleral depression can be traumatic, because the eye is distorted and the intraocular pressure (IOP) is transiently elevated. However, the effect of scleral depression on the IOP has not been evaluated quantitatively.

Thus, the purpose of this study was to evaluate quantitatively the effects of the IOL diameter on the IOP induced by scleral depression to observe the peripheral fundus. To accomplish this, we implanted a pressure transducer in the vitreous and, also, implanted IOLs into enucleated porcine eyes. The IOP was then measured during scleral depression.

## 2. Materials and Methods

**Figure 1:**
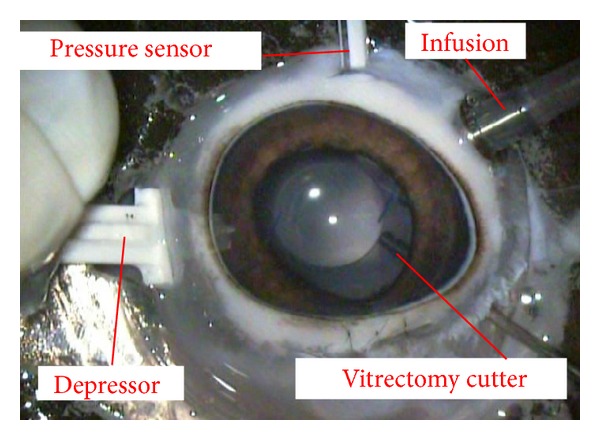
Photograph of enucleated porcine eye showing the experimental setup. After combined pars plana vitrectomy, lens aspiration, and IOL implantation, a pressure sensor was introduced into the vitreous cavity through a sclerotomy at 6 o'clock. Scleral depression was performed at 10 mm posterior from the corneal limbus at 3 or 9 o'clock.

**Figure 2:**
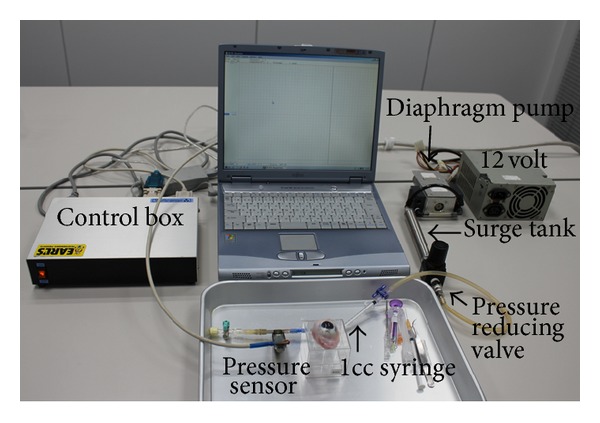
A custom made system to deliver constant pressure. Constant pressure (40 mm Hg) was delivered to the inner cylinder of a disposable 1cc syringe, which was used as a scleral depressor. And the external cylinder was held so that the tip of the inner cylinder was just touching the sclera 10 mm posterior from corneal limbus by surgeons.

Combined pars plana vitrectomy, lens aspiration, and IOL implantation were performed on 4 enucleated porcine eyes (Figure 1). We implanted either the eternity X-60 or the eternity X-70 IOLs (Santen Pharmaceuticals, Osaka, Japan) into the porcine eyes. The diameter of the optics was 6.0 mm in the X-60 and 7.0 mm in the X-70 IOL. After the core vitreous was removed, a pressure transducer (Portable, 2-Channel, Serial Port I/O Module, Motorola Solutions Japan Ltd, Tokyo, Japan) was placed into the midvitreous through a sclerotomy at 6 o'clock. Scleral depression was performed at 10 mm posterior from corneal limbus at 3 or 9 o'clock. Constant pressure was delivered to the inner cylinder of a disposable 1cc syringe. And the external cylinder was held by surgeons (Figure 2). Physiological salt solution was used for the infusion solution, and the bottle height of the infusion was kept at 40 cm above the eye. To minimize the influence of the pupil diameter on the peripheral retinal observation through the optics, enucleated porcine eyes with similar pupil diameter was used. The apparent pupil diameter (mm) through cornea was 10.92 and 11.01 in eyes with X-60 and 10.96 and 10.98 in eyes with X-70.

The intraocular pressure (IOP) was monitored during scleral depression, in eyes with X-60, 9 times and, in eyes with X-70, 8 times. The IOP was measured from the beginning of the indentation to the endpoint when the peripheral retina was visible through the IOL optics. Then the IOP values were plotted as a function of time, and the area under the curve was determined.

### 2.1. Statistical Analyses

The AUC during scleral depression and the time from the beginning to the endpoint when the peripheral retina were visible through the IOL optics, and the IOP elevation at the endpoint was measured, unpaired *t* tests were used to determine the significance of the differences of these parameters between eyes with the eternity X-60 and the eternity X-70. A *p* < 0.05 was taken to be significant.

## 3. Results

**Figure 3:**
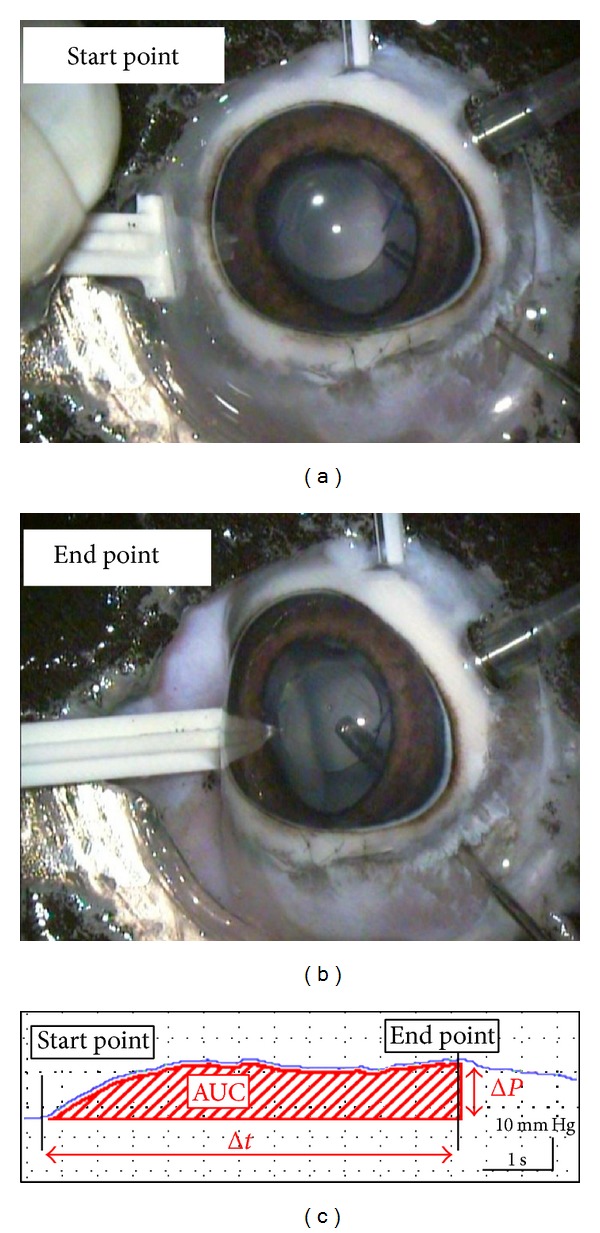
Representative graph showing IOP changes during scleral indentation in eye with X-60 implanted. (a) Scleral depression was performed at 10 mm posterior from the limbus at 3 or 9 o'clock. (b) Scleral depression was performed so that the peripheral retina can be seen through the optics of X-60 implanted in the anterior chamber. The markings of the graph show that the starting (arrowhead) and end (arrow) points of the scleral depression are shown in the lower figure. (c) Graph of the intraocular pressure (IOP) as a function of time during the scleral indentation. The area under the curve (AUC) of the IOP from the beginning of the indentation (start point) to the endpoint when the peripheral retinal surface was observed through the IOL was measured. Abscissa axis: time (0.5 seconds/scale), vertical axis: intraocular pressure (10 mm Hg/scale).

**Figure 4:**
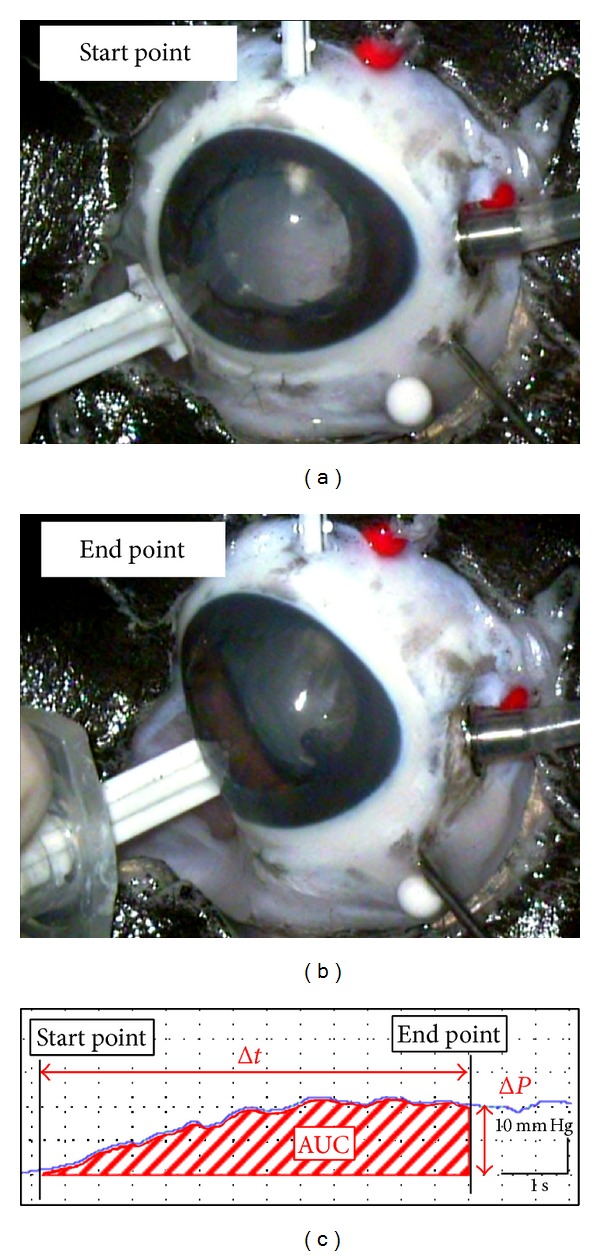
Representative plot of the IOP changes during scleral indentation in eye with X-70 IOL implanted. (a) Scleral depression was performed at 10 mm posterior from corneal limbus at 3 or 9 o'clock. (b) The scleral indentation was performed so that the peripheral retina can be seen through the optics of a X-70 IOL implanted in the anterior chamber. The markings of the plotting show that the starting (arrowhead) and end (arrow) points of the scleral indentation are shown in the lower figure. (c) Graph of the IOP as a function of time. The area under the curve (AUC) of the IOP from the beginning of the indentation to the endpoint when the peripheral retinal surface was observed through the IOL optics was measured. Abscissa axis: time (0.5 seconds/scale), vertical axis: intraocular pressure (10 mm Hg/scale).

Representative graphs of the IOP change during sclera depression of the eye are shown in Figures 3 and 4. The mean ± standard error of the means (SEM) AUC was 908.5 ± 244.1 mm Hg sec in eyes with the eternity X-60 and 303.5 ± 40.2 mm Hg sec in eyes with the eternity X-70 (*p* < 0.01, Table 1). The time from the beginning to the endpoint when the peripheral retina was visible was 9.8 ± 2.1 sec in eyes with the X-60 and 7.3 ± 1.1 sec in eyes with the X-70 IOL (*p* = 0.29, Table 1) during the sclera depression when great care was taken to exert uniform pressure. The IOP elevation at the endpoint was 50.5 ± 4.5 mm Hg in eyes with the X-60 and 37.0 ± 1.9 mm Hg in eyes with the X-70 (*p* < 0.01, Table 1).

## 4. Discussion

Scleral depression is used to examine the peripheral retina and also during the shaving of the peripheral vitreous, endolaser photocoagulation of the peripheral retina, and dissection or removing of peripheral membranes. Although endoscopy can be used to avoid scleral indentation, it requires experience and skill. Another solution to the stress on the eye by scleral depression may be to perform the IOL implantation after the vitrectomy or selecting a large diameter IOL [5, 6]. Several authors have reported that an IOL with relative large diameter was advantageous, because the peripheral fundus was visible during the vitreous surgery [5, 6]. With smaller diameter IOLs, the fundus is viewed sometimes through the optics and other times around the optics. Thus, there is a need for continuous refocusing of the operating microscope.

The effect of the IOL diameter on the changes in the IOP induced by scleral depression had not been evaluated. Our results showed that the AUC was smaller and the time to reach the endpoint was shorter with the larger diameter IOL during scleral depression. This is probably because the distance between the peripheral retinal surface and the edge of the IOL optics is farther in eyes with the smaller diameter IOL, and, therefore, a deeper indentation is necessary to bring the peripheral retina into view through the IOL optics. Our results suggest that larger diameter IOL may be able to reduce the depth of scleral indentation, thereby, minimizing the stress on the eye during scleral depression.

Our findings suggest that the AUC may be used to determine the degree of stress on the eye during scleral depression. The system may also be used for surgeon's exercise for scleral depression.

There are some limitations in our study. Scleral depression was performed with great care to exert uniform pressure. But the speed and pressure depended on the surgeon's experience. The results showed that Δ*P* was significantly different between eyes with the X-60 and eyes with the X-70 IOL but Δ*t* was not significantly different. This might suggest that the speed rather than the pressure was constant during depression. Constant pressure during scleral indentation by using a syringe pump like system would enable more precise comparison of the IOP change between each indentation. Due to the study design, the surgeon could not be masked to the type of IOL implanted. In addition, the IOP elevation during scleral indentation is influenced by several factors, such as the size of the eye, the rigidity of the eye wall, the position of the indentation, and the material filling the vitreous cavity. We believe that similar results can be observed in the clinical setting, because the size and rigidity of the eye of the porcine eye are not so different from the human eye. In our experiment, the scleral depression was done at 10 mm posterior from the limbus and the IOP change during indentation was measured after core vitrectomy. These procedures are similar to the clinical situation and care was taken to keep these factors constant. It would be interesting to measure the IOP change during scleral indentation when the vitreous cavity is filled with other materials such as air, silicone oil, and perfluorocarbon liquid or during fluid/air exchange.

Recently introduced vitrectomy system (Alcon Constellation Vision System) is equipped with a pressure control system and can maintain IOP at constant, independent of aspiration flow rates during vitrectomy. The IOP control system takes several seconds during the process of IOP detection and feedback [7]. Our system measures the IOP directly and does not have such an inherent time lag. Sugiura et al. reported that IOP rapidly increased to 70–100 mm Hg and then slowly decreased to 30 mm Hg in 3.5–4.0 seconds during scleral depression without aspiration, with or without the IOP control system. If the IOP change against the time can be plotted, the AUC may be used as a parameter reflecting the degree of stress of the eye during vitrectomy maneuvers.

In conclusion, we were able to measure the IOP during scleral depression in enucleated porcine eyes implanted with different diameter IOLs. Analysis of the IOP changes clearly showed that an IOL with larger optics can minimize the effects of the scleral indentation.

## Figures and Tables

**Table 1:** Comparisons of the scleral depression parameters between eye with X-60 and X-70.

IOL size	AUC	Δ*t*	Δ*P*
X-60	908.5 ± 244.1	9.8 ± 2.08	50.5 ± 4.51
X-70	303.5 ± 40.2	7.26 ± 1.11	37.0 ± 1.92
*p*	*p* < 0.01	*p* = 0.29	*p* < 0.01

Each value is shown as mean ± S.E.

IOL: intraocular lens, AUC: area under the curve.
